# Large field-of-view event-mode camera for high-precision epithermal neutron resonance imaging

**DOI:** 10.1038/s41598-025-96789-8

**Published:** 2025-04-23

**Authors:** Tsviki Y. Hirsh, Alexander M. Long, Adrian S. Losko, Tim T. Jaeger, Alexander Wolfertz, Sven C. Vogel

**Affiliations:** 1 Soreq NRC, Yavne, 81800 Israel; 2 Los Alamos National Laboratory, Los Alamos, New Mexico 87545 United States of America; 3 Forschungs-Neutronenquelle Heinz Maier-Leibnitz, Garching, 85748 Germany; 4 Technische Universität Darmstadt, Darmstadt, 64289 Germany

**Keywords:** Imaging techniques, Experimental nuclear physics

## Abstract

A large-area event-mode camera system coupled with a $^{6}$LiF-ZnS:Ag scintillator is applied for neutron resonance imaging (NRI) on the energy-resolved neutron imaging (ERNI) flight path, also known as Flight Path 5 (FP5), at the Los Alamos Neutron Science Center (LANSCE). This novel neutron imaging system, featuring a 120$\times$120 mm$^2$ field of view, efficiently captures resonance information across the entire image in a single acquisition, significantly reducing beam time requirements compared to conventional energy-resolved neutron imaging systems. High-quality neutron radiographs with enhanced spatial resolution are achieved through the reconstruction of neutron events based on observations of individual photons emitted from the scintillator. The system demonstrates reduced background through neutron/gamma discrimination capabilities while maintaining sharpness across a large fields of view. In the measurements presented here, a spatial resolution of approximately 340 $\mu$m was achieved using center-of-gravity photon cluster centroiding. We demonstrate the system’s capability for quantitatively determining isotopic distributions in various thin samples, as well as automatically reconstructing complex scenes with overlapping resonances from diverse samples. These results are obtained using standard data analysis tools, despite the relatively slow $^{6}$LiF-ZnS:Ag scintillator, which may not be optimal for absorption resonance detection. The capabilities demonstrated here offer a valuable, versatile, and cost-effective solution for high spatial and temporal resolution, large field-of-view energy-resolved neutron imaging, with potential applications across various scientific and industrial domains.

## Introduction

Neutron imaging has emerged as a preeminent non-destructive technique, offering unique insights into the internal structures and properties of materials. This is due to the interaction of neutrons with nuclei in the material, rather than the electronic shell as is the case for protons, electrons, and photons. Its exceptional penetrating capabilities and sensitivity to nuclear structure make neutron imaging a powerful tool across diverse scientific disciplines, including material characterization, engineering, and nuclear applications^1–4^. Neutron resonance imaging (NRI) stands out as a novel approach that leverages the unique signatures of nuclear reaction cross-sections within the resolved-resonance region. This technique enables identification and quantification of specific isotopes in complex material compositions, such as geological samples^5^, scintillator materials^6^, or nuclear fuels^7,8^. The detection system used for NRI in these studies consisted of a boron-doped microchannel plate (MCP) coupled with the TimePix sensor^9^. The combination of a neutron-sensitive MCP and the TimePix sensor allows high-speed frame rates, enabling sufficient data sampling of resonances on a pixel-by-pixel basis. However, despite its high efficiency, certain limitations-such as its relatively high sensitivity to gamma backgrounds^7^-posed challenges for quantitative NRI measurements and analysis. Furthermore, the system has practical usability limitations. The MCP detector’s high vacuum and voltage requirements increase the complexity of the experimental setup. Additionally, the small field of view (FoV) of 28$\times$28 mm$^{2}$ restricts measurements to smaller samples or necessitates multiple acquisitions and sample manipulations to assemble complete image stacks.

Recently, the commercial availability of the Timepix3 sensor with innovative data-driven readout systems has marked a significant technological leap. Unlike traditional frame-based sensors, Timepix3 pixels operate independently and, when triggered, output data such as pixel position, time of arrival (ToA), and time over threshold (ToT) into a continuous data stream. These advancements have led to the development of a novel neutron camera system, the LumaCam, which employs optically sensitive Timepix3-based sensors coupled with neutron-sensitive scintillation screens (such as $^{6}$LiF-ZnS:Ag) via image intensifiers and optical lenses. The LumaCam presents several advantages that address the limitations of the current MCP-TimePix imaging system. Instead of relying on specialized boron-doped MCPs to perform the neutron capture, LumaCam employs conventional neutron-sensitive scintillators. While LumaCams still use MCPs, they are incorporated as part of the image intensifiers, which are off-the-shelf components with robust integrated solutions for vacuum and high voltage, requiring little to no user intervention. Furthermore, by optically coupling the scintillator to the image intensifier via lenses, LumaCams achieve significant variation in the in system’s FoV.

Certain classes of scintillators exhibit different scintillation responses to neutrons and photons. This property can be used to reject gamma radiation background, which is often observed in neutron imaging. This contrasts with MCP detectors, where the produced avalanche is less sensitive to differences between neutrons and gamma radiation. Moreover, LumaCam detectors demonstrate effective centroiding capabilities, similar to those of MCP-TimePix2 systems^10^, enabling resolution enhancement beyond the native pixel size. Losko et al.^11^ demonstrated substantial improvements in thermal and cold neutron imaging using a LumaCam detector coupled with a $^{6}$LiF-ZnS:Ag scintillator. However, the benefits of using these systems with epithermal neutrons for resonance imaging have not yet been explored.

In this study, we extend the capabilities of the LumaCam system by demonstrating its effectiveness in producing detailed neutron images using epithermal neutrons. This capability allows neutron radiography of materials that strongly absorb thermal neutrons, such as fissile materials. Additionally, we leverage the system’s high timing resolution to apply time-of-flight (TOF) techniques for measuring neutron absorption resonances. This enables precise identification and quantification of isotopic densities in the examined samples.

## Experimental setup

### Facility and beam configuration

The measurements were conducted at the energy-resolved neutron imaging flight path (ERNI), also known as flight path 5 (FP5)^12^, a pulsed thermal/epithermal neutron imaging instrument at the Lujan Neutron Scattering Center^13^ at the Los Alamos Neutron Science Center (LANSCE). ERNI/FP5 has two distinct detection stations. The first station, located at a shorter path length of $6.5-11$ meters, was used for this experiment due to its high neutron flux. The second station, positioned at $58-61$ meters, is optimal for measurements requiring low background or high neutron TOF resolution. The beam was divergently collimated within the evacuated incident beam pipe over a 2-meter distance with an inner diameter of 2 cm. To further reduce background, a beam scraper consisting of circular annular discs, each with alternating 5 cm layers of steel and high-density polyethylene, along with a central steel pipe with a 2 cm internal diameter, was used. To mitigate the gamma flash emitted from the target, a 5 cm thick lead brick was placed at the beam entrance of the first station. With a collimator-to-detector distance of approximately 4 meters and a beam diameter of 2 cm, the resulting collimation ratio (L/D) was approximately 200. The LANSCE accelerator delivers 800 MeV protons at a frequency of 20 Hz, with a base-to-base proton pulse width of 250 ns, to the tungsten spallation target at the Lujan Center^14^. The moderator for ERNI/FP5 consists of a 35 mm thick water container at 25 $^\circ$C^15^, providing high flux and medium resolution^16^.

### Neutron camera setup

**Fig. 1:**
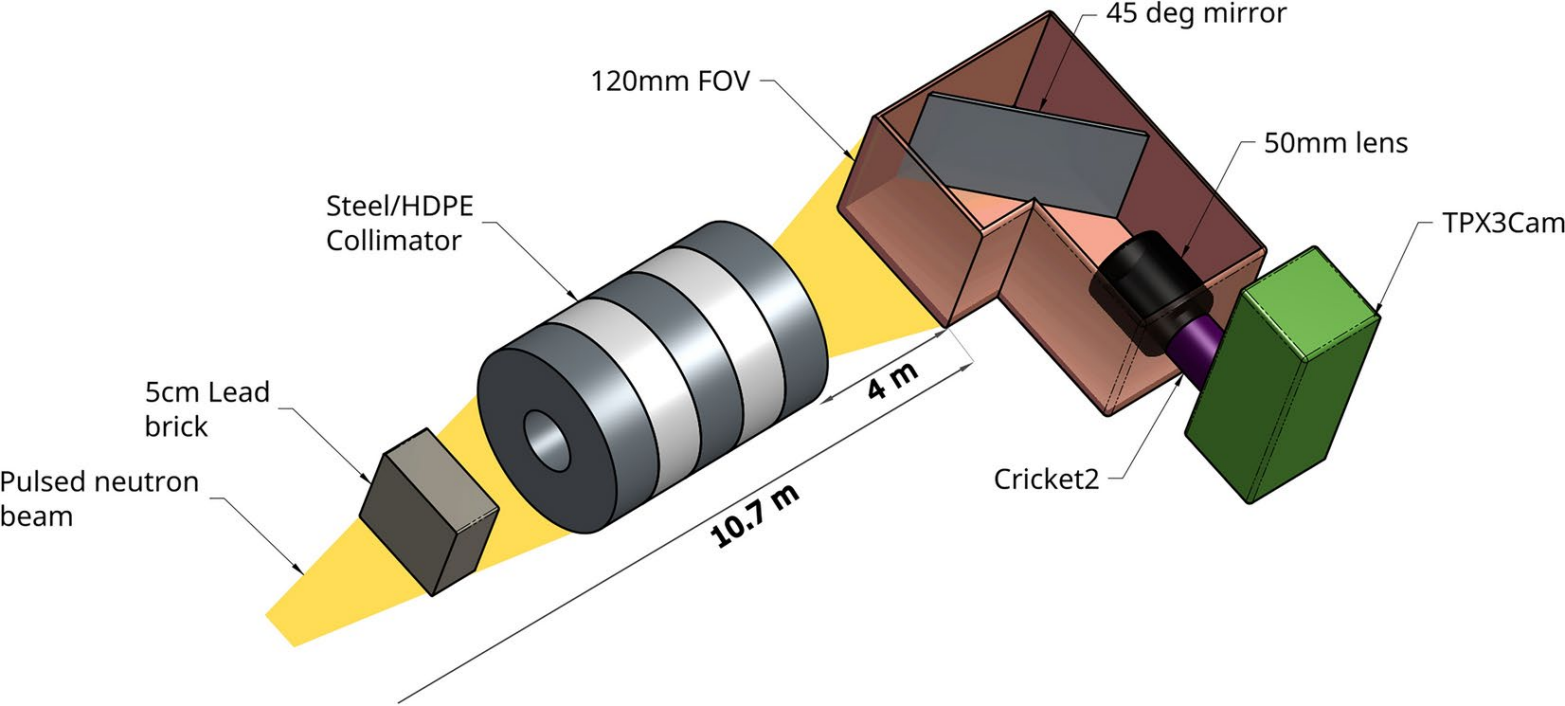
Schematic of the large FoV LumaCam setup at ERNI/FP5 instrument (not to scale). The pulsed neutron beam traverses a 5 cm Pb block and collimator before reaching the LumaCam, 10.7 m from the moderator. Neutrons are converted to optical photons on a 120$\times$120 mm scintillator screen, relayed and intensified through optics, and detected event-by-event using a TPX3Cam.

The LumaCam detector is positioned 10.7 meters downstream of the moderator, with the scintillator screen serving as the neutron-sensitive component that defines the flight path length, as shown in Fig. 1. The scintillator is a 200 µm thick $^6$LiF-ZnS:Ag screen (see Section 2.3 for details). The scintillation light from this screen is reflected by a 45-degree mirror and focused through a lens onto a dual-stage, chevron-type image intensifier^17^. The gain input of the image intensifier was set to 1 V, corresponding to an estimated gain of 10$^5$, with a quantum efficiency of approximately 30% for photon-to-charge conversion in the light-sensitive photocathode. The gating of the image intensifier was synchronized with the accelerator’s trigger, operating at a frequency of 20 Hz with a gate duration of 2.5 ms. No delay was applied, thereby capturing events from the gamma flash of the spallation process. This configuration allows detection of neutrons in the relevant energy range of $0.1-300$ eV. The intensified image is then relayed through another lens to the TPX3Cam^18^, which is equipped with an integrated Timepix3 sensor.

Data is streamed from the sensor via a SPIDR readout chip and a high-speed 10 Gb/s fiber-optic connection, which is directly linked to a data acquisition computer^19^. For each triggered pixel, the Timepix3 sensor records the timestamp for ToA, with a precision of 1.56 ns, along with the ToT information and the x and y coordinates of the hit pixel. Once a pixel is triggered above the threshold, the readout time (or per-pixel dead time) is 475 ns. The inherent data-driven readout capabilities of the Timepix3 sensor and SPIDR board enable advanced event reconstruction algorithms.

The signal processing algorithm follows a two-stage clustering approach, where individual pixel activations are first grouped into scintillation photons, and subsequently, these photons are clustered into neutron events. This hierarchical reconstruction method enables high spatial and temporal accuracy in event detection. The two-stage reconstruction approach was first introduced in^11^ and has since been formalized and described in detail in^20^. The parameters used for event reconstruction are summarized in Table 1.

**Table 1:** Reconstruction parameters used for the two-stage clustering process based on methods described in Losko et al.^11^ and Wolfertz et al.^20^.

Pixels to Photons			Photons to Events		
Parameter	Symbol	Value	Parameter	Symbol	Value
Maximum spatial separation	$d_{\text {px},s}$	1 px	Maximum spatial separation	$d_{\text {ph},s}$	3 px
Maximum temporal separation	$d_{\text {px},t}$	50 ns	Maximum temporal separation	$d_{\text {ph},t}$	100 µs
Minimum pixel activations	*k*	2	Maximum event duration	$D_t$	500 µs
			Minimum photon number	*m*	3

The first stage groups pixel activations into scintillation photons, while the second stage clusters photons into neutron events. Consistent parameters for spatial and temporal separation are used at each clustering stage.

In the first stage, pixel activations are clustered to reconstruct individual scintillation photons. This clustering is performed using a hierarchical single-linkage method, where a pixel is assigned to a cluster if it is within a maximum spatial separation of $d_{\text {px},s}$ and a maximum temporal separation of $d_{\text {px},t}$ from another activated pixel. To be considered a valid scintillation photon event, each cluster must contain at least *k* activated pixels. The centroid of the cluster, weighted by the time-over-threshold (ToT) of each activated pixel, determines the reconstructed photon position. The arrival time of the first pixel activation within the cluster is assigned as the photon detection time.

In the second stage, reconstructed scintillation photons are clustered to identify neutron interaction events. This stage also employs hierarchical single-linkage clustering, grouping photons if they are within a maximum spatial separation of $d_{\text {ph},s}$ and a maximum temporal separation of $d_{\text {ph},t}$. The total event duration is restricted to $D_t$, ensuring that photon clusters spanning longer durations are split to prevent multiple neutron events from being merged into a single cluster. To be classified as a neutron event, each cluster must contain at least *m* photons. The reconstructed neutron position is determined by computing the center of gravity of the clustered photons, calculated as the mean of their spatial coordinates, while the neutron event timestamp is assigned based on the arrival time of the earliest photon in the cluster.

The final set of reconstructed neutron events is binned into three-dimensional images with dimensions of (2000, 512, 512), where the first axis represents TOF in 1 µs bins and the remaining two axes correspond to sub-pixel spatial dimensions. This event-based clustering approach significantly enhances neutron/gamma discrimination and improves spatial and temporal resolution in neutron imaging.

### Scintillator screen

In neutron imaging, the scintillator screen plays a crucial role in converting incoming neutrons into detectable light signals. The choice of scintillator material significantly impacts the experiment’s success by influencing factors such as spatial resolution, temporal resolution, and neutron-gamma discrimination. This directly impacts the signal-to-noise ratio of the acquired image. For our setup, which features a 120$\times$120 mm$^2$ FoV and an 80$\times$100 mm$^2$ beam spot illumination area, we selected a 200 µm thick $^{6}$LiF-ZnS:Ag scintillator screen from rcTRITEC ^21^. This choice was motivated by several key factors. First, its high light output of approximately 150,000 optical photons per neutron capture enables efficient neutron detection^22^. Additionally, the scintillator produces approximately 75,000 photons per gamma event, allowing for excellent neutron-gamma discrimination^22^. This discrimination capability improves the signal-to-noise ratio in neutron imaging. However, there exists a trade-off between high light output and decay time. While $^{6}$LiF-ZnS:Ag provides a high light yield, it has a moderately long decay time of 5 and 80 µs^23^. This can limit the achievable count rate and increase system dead time, especially for high-flux applications. Future investigations into alternative scintillators with faster decay times may be beneficial for such scenarios. However, in this work, prioritizing established parameters for neutron event identification was crucial. Using the same scintillator screen as in previous experiments ^11^ minimized the time required to optimize neutron event reconstruction routines.

Lastly, a key consideration in our setup is the interaction probability of epithermal neutrons with the scintillator screen, which is limited by the screen’s thickness. For our 200 µm thick screen, the estimated interaction efficiency for 1 eV epithermal neutrons is approximately 0.1%. This low efficiency is a known challenge in scintillator-based imaging systems for epithermal neutrons. While increasing the screen thickness could enhance the interaction probability, it may also compromise spatial resolution. This trade-off between efficiency and resolution is an crucial factor when applying this setup to epithermal neutron imaging.

### Sample and measurement configurations

To demonstrate the system’s capabilities, we performed three types of measurements. The first measurement involved 1 mm thick tungsten and tantalum sheets that covered the entire beam and were positioned 6.7 m from the moderator, at the downstream end of the collimator, and 4 m upstream of the camera. The strong neutron absorption cross-sections of these materials at specific resonance energies allow for the characterization and quantification of background contributions within the setup. Additionally, this enables direct comparison with background contributions reported in previous MCP-TimePix measurements performed on ERNI/FP5. For the second measurement, we remeasured various samples previously analyzed in NRI experiments conducted on ERNI/FP5 using the established MCP-TimePix2 detector^5–7,24^. Taking advantage of the substantially larger FoV (120 $\times$ 120 mm$^2$) compared to the MCP-TimePix2 detector (28 $\times$ 28 mm FoV), we arranged a collage of these samples to demonstrate the improved qualitative material identification capabilities with the LumaCam. The third measurement involved thin foils of iridium (50 $\mu$m thick), tantalum (25 $\mu$m thick), and gold (20 $\mu$m thick), partially covering the scintillator screen. These foils were affixed using aluminum tape near the aluminum backing of the scintillator screen and intentionally overlapped to create stacks with varying numbers of layers. This arrangement facilitated the evaluation of isotope quantification capabilities using NRI techniques and enabled the assessment of the overall spatial resolution of this type of LumaCam setup (large FoV coupled with a 200 µm $^{6}$LiF-ZnS:Ag scintillator).

### Data analysis

Typically, for each measurement, a region of interest (ROI) was selected from the time-resolved images of both the sample and corresponding open beam measurements, summing all counts across pixels within the ROI. Since the LumaCam is a counting detector following Poisson statistics, we calculated the energy-dependent transmission, $T(E_n)$, as $T=S(E_n)/O(E_n)$, where $S(E_n)$ represents the sample count spectrum and $O(E_n)$ denotes the open beam count spectrum. Uncertainty was estimated under the assumption of Poisson statistics.

To extract quantitative isotopic measurements from the measured transmission data, we adopted SAMMY^25,26^, a well-established and versatile open-source R-Matrix code widely used in nuclear data evaluations. Although SAMMY is typically used for analyzing thin, uniform samples with conventional mono-pixel neutron detectors, we adapted it for our specific analysis using the Python code PLEIADES^27^. SAMMY applies Bayes’ theorem to fit a theoretical model to transmission data, which which follows the exponential decay described by the Beer-Lambert law: $T(E_n) = e^{-n \sigma (E_n) d}(1 - B) + B$, where $\sigma (E_n)$ is the total cross-section, *n* is the atomic weight density in atoms/barn-cm, *d* is the average sample thickness in cm, and *B* is the background-to-open beam ratio ranging from 0 to 1. For SAMMY fitting in this work, we used a constant term, $B_{const}$, to describe the background, which was sufficient for accurate characterization. Resonance parameters for various isotopes were obtained from ENDF-VIII.0^28^. During the fitting process, resonance parameters were fixed while the isotope areal density parameter was varied to determine the average sample thickness in each ROI.

While SAMMY is a powerful tool, it has certain limitations. One major limitation is its statistical requirement. Accurate fitting of isotopic densities with SAMMY relies on high-quality transmission data with sufficient counts, which becomes challenging as ROIs approach the native pixel pitch. Additionally, the computational time required for SAMMY to converge on a solution can be substantial, especially for complex samples with multiple isotopes. This can create a bottleneck in processing large datasets or performing real-time analysis.

To address these challenges and optimize transmission spectra fitting on a native pixel resolution, the open source NRI analysis code, TRINIDI^29^, was developed at Los Alamos National Laboratory (LANL). TRINIDI employs a two-step process that first estimates neutron flux and background counts, then reconstructs the areal densities of each isotope at the pixel level. By inverting a forward model that accounts for non-linear absorption, energy-dependent emission profiles, Poisson noise, and spatiotemporal variations in background and flux, TRINIDI enables accurate 2D and 3D isotope density reconstructions. Notably, TRINIDI is orders of magnitude faster in computational time than SAMMY, significantly improving the efficiency of data processing. Therefore, we employ TRINIDI to analyze the larger FoV NRI measurements in this work.

For both SAMMY and TRINIDI analyses, we used a modified instrument response function derived from a previously calibrated functional form developed at ERNI/FP5^30^. This instrument response function includes shape parameters ($v_1$, $v_2$), time constants ($T_1$, $T_2$), shift parameters ($t_1$, $t_2$), and a weighting factor ($w_1$) between the two time-distribution components. To account for the longer decay characteristics of the $^6$LiF-ZnS:Ag scintillator, we adjusted the response function parameters to fit our measurements, tuning only $t_1$ to 2.5 $\mu$s while keeping the remaining parameters identical to those originally measured for the ERNI/FP5 instrument response: $v_1=6$, $v_2=4.3$, $w_1=0.65$, $T_1=0.74~\mu$s, $t_2=2.2~\mu$s, and $T_2=5.1~\mu$s.

## Results

### Background determination

**Fig. 2:**
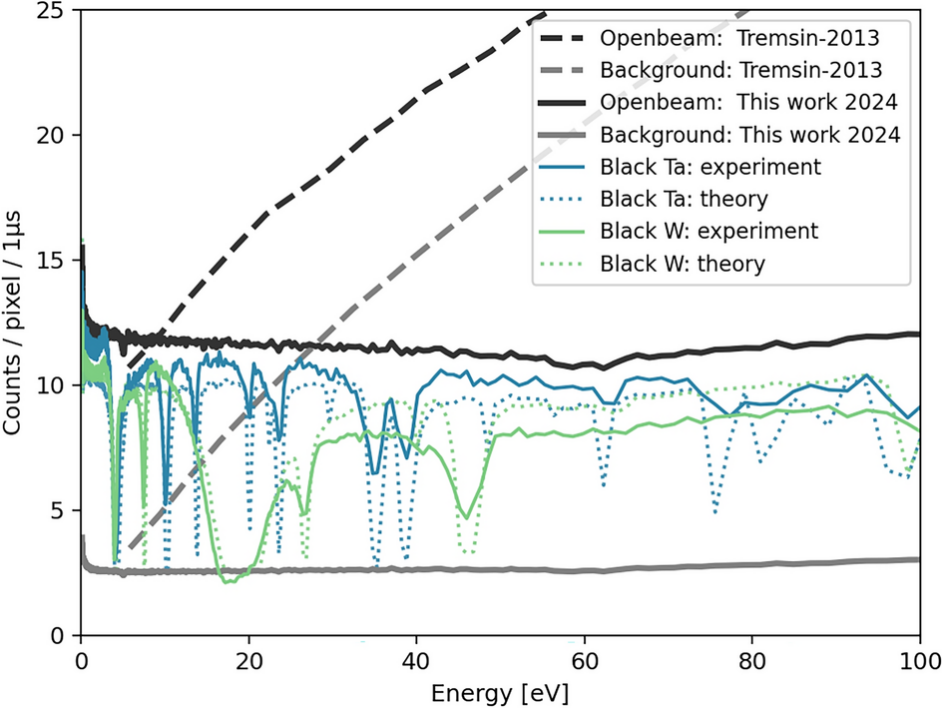
Estimated background levels from “black” resonance measurements using thick W and Ta foils compared to open beam intensities for this work and a previous study^7^. Experimental results are shown along with theoretical calculations.

We assessed the background level and calibrated the effective flight path length using the well-established “black” resonance technique^31^ applied to data collected with 1 mm tungsten and tantalum sheets. Each measurement, including the open beam measurement, lasted approximately $3-5$ hours, with a total proton charge of 18.5 mA-h. The measured resonance spectrum results from the convolution of the theoretical transmission spectrum with the instrument response function. The extended decay time of the ZnS:Ag scintillator causes the detection system’s resolution function to broaden and reduce the depth of the observed resonance features compared to theoretical values, as illustrated in Fig. 2. As a result, the resonance saturation is less pronounced than expected. The background level was determined by optimizing the fit between a background function and the theoretical cross-section of the black resonance materials. However, strong distortions in resonance shapes, particularly near broad and saturated features, along with the broad instrument response, make it difficult to visually align the background curve precisely with the base of the black resonances. Despite these challenges, our approach yields the best possible estimate of the background level. Notably, the background level of our system is significantly lower than that of the boron-doped MCP technology previously used at ERNI/FP5. To illustrate this, Fig. 2 includes dashed lines representing the previously observed background and open beam intensities from reference^7^. Both measurements were conducted at the same beamline with similar black resonance samples under comparable environmental conditions. To our understanding, the observed difference in background levels primarily results from the improved gamma-neutron discrimination and suppression capabilities of our detector compared to MCP-based technology. This enhancement reduces background and improves the signal-to-noise ratio.

### Spatial resolution

**Fig. 3:**
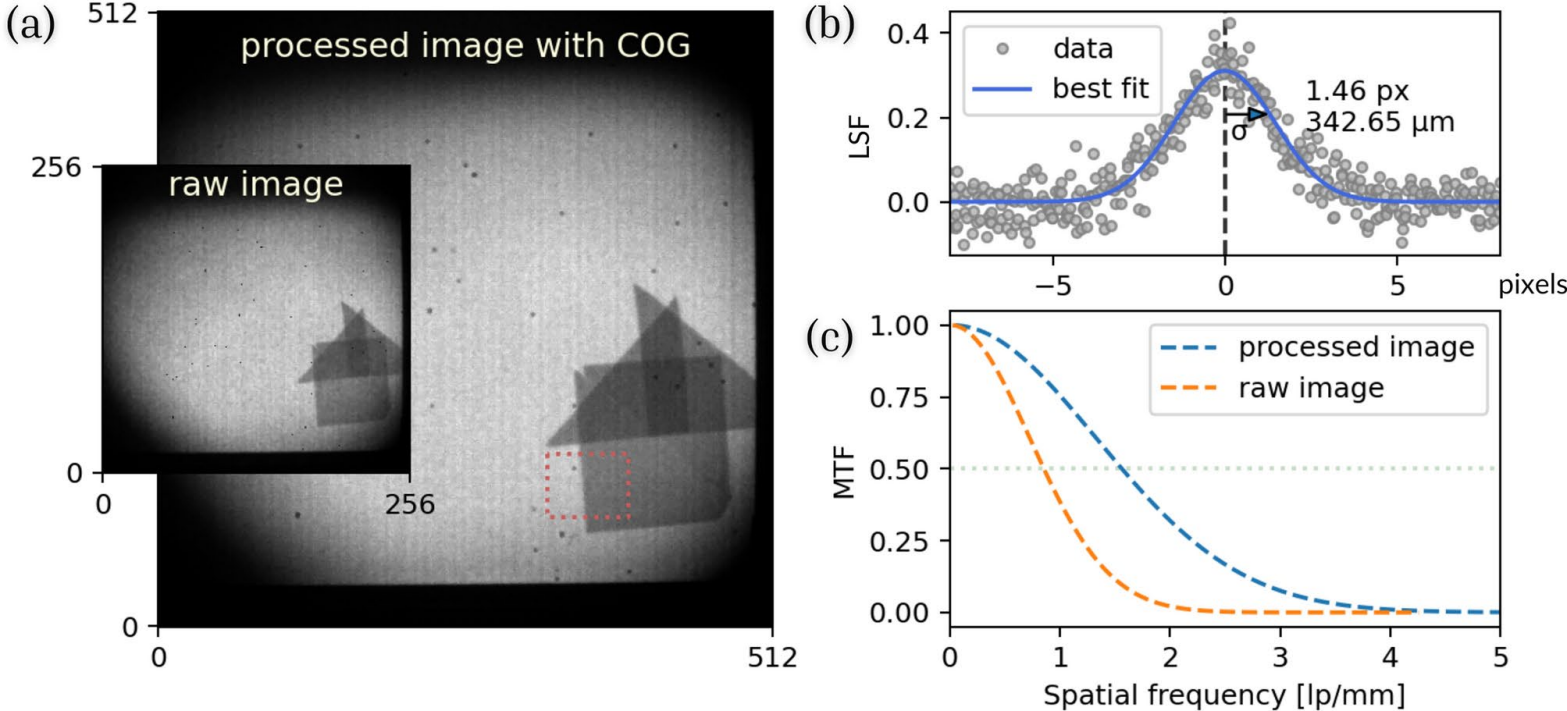
Analysis for resolution estimation: (**a**) Comparison of the raw neutron and the processed transmission images with COG algorithm applied. (**b**) Gaussian fit of the gradient across the ROI marked by the dashed line in the processed image, indicating a resolution of 343 µm. (**c**) The corresponding modulation transfer functions (MTF) for both the raw image and the processed image, demonstrating the improved resolution in the processed image.

The system’s spatial resolution was determined using modular transfer function (MTF) analysis, which requires high-contrast, sharp-edged objects positioned at an angle to the sensor’s pixel array. For this MTF analysis, data recorded with multiple thin foils of different materials in the beam was used. An edge of one of the iridium foils, measured at the lowest energy absorption resonance of iridium, was used as the sharp edge. Figure 3a shows the raw transmission image of pixel hits on the 256$\times$256-pixels Timepix3 sensor as well as a processed 512$\times$512-pixel image created using the COG algorithms, as discussed above. The ROI for MTF calculation is indicated by a dashed line. The MTF was calculated as follows: First, Gaussian functions were fitted to the row-wise derivative of the observed pixel intensities across the transition edge to estimate the line spread function (LSF) of the imaging system (see Fig. 3b). The width of these Gaussian curves serves as an estimate of the system’s spatial resolution. The LSF fit curve was then Fourier transformed to obtain the MTF (shown in Fig. 3c). The resolved frequency, measured in line pairs per millimeter (lp/mm), corresponds to the spatial frequency where the MTF curve reaches 50% ^32^. The Gaussian fit for the processed data has a standard deviation of 1.46 pixels, corresponding to a spatial resolution of approximately 343 µm across the 120$\times$120 mm FoV. The raw image and the processed neutron image obtained through our COG algorithm exhibit MTF curves reaching 50% at approximately 0.9 and 1.6 lp/mm, respectively. This indicates nearly a twofold improvement in spatial resolution after processing. The achieved resolution of 340 µm is consistent with the results reported for a similar system using thermal neutrons ^11^.

### Automated reconstruction of isotopic densities of complex samples

Real-world samples often contain multiple materials with non-uniform compositions and irregular shapes, posing challenges for NRI and subsequent analysis. To evaluate the limits of material identification and quantification in realistic NRI measurements, we created a complex object scene using a large-FoV NRI setup coupled with a $^{6}$LiF-ZnS:Ag scintillator screen.

**Fig. 4:**
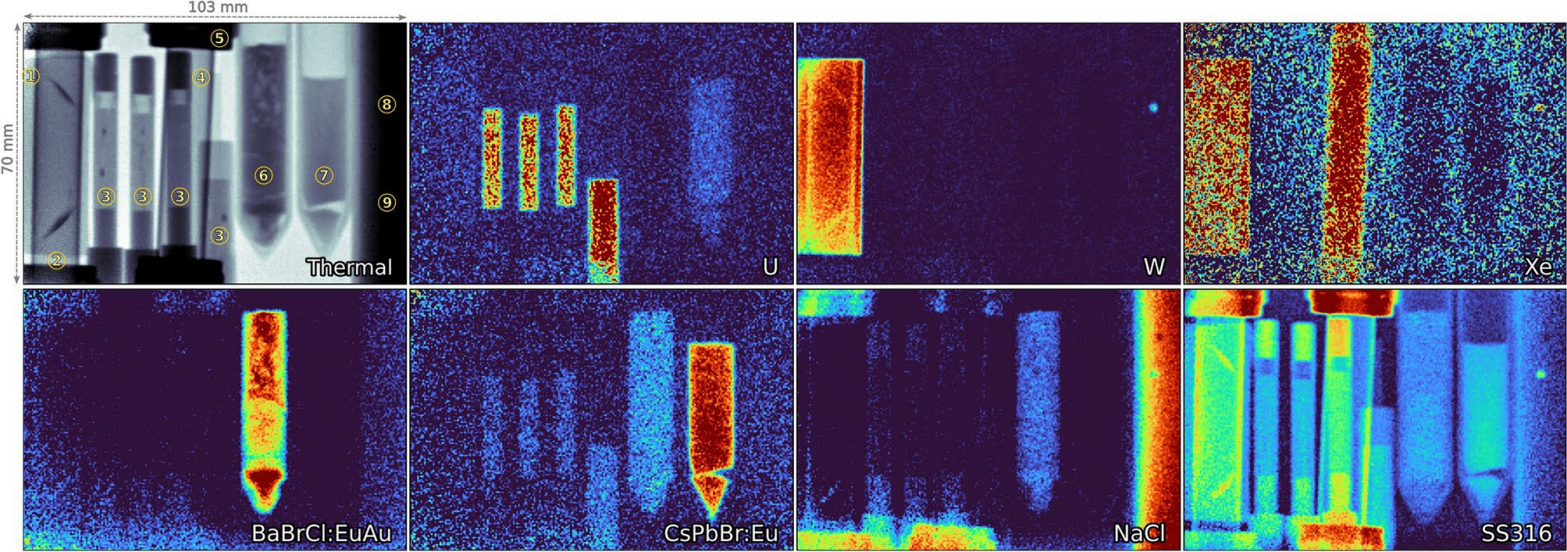
(Top left) Thermal neutron radiograph of a 70$\times$103 mm$^2$ ROI covering the sample collage. (Other images) TRINIDI-based reconstructions of areal density for different materials, with warmer colors indicating higher densities. Samples include: (1) tungsten foil (W), (2) krypton gas cell (Kr), (3) depleted uranium oxide fuel pellet assemblies (U), (4) xenon gas cell (Xe), (5) Swagelok SS316 tube caps, two scintillators: (6) BaBrCl:EuAu and (7) CsPbBr$_3$:Eu, (8) 2” silica glass tube filled with NaCl powder, and (9) three small beads (borosilicate glass, zirconium, and tungsten carbide) embedded within the powder.

As shown in Fig. 4 and marked with circled numbers, the samples were arranged side by side directly in front of the scintillator screen. Ordered from left to right, a 2-mm-thick tungsten square sheet (1) was placed first. Next to it were four distinct assemblies of depleted uranium dioxide fuel pellets ^7^ (3). Each assembly consisted of low-enrichment pellets (4.6 mm in diameter) clad in aluminum, with varying densities and inclusions, such as tungsten wires or void inserts. However, due to the spatial resolution limitations of our large-FoV imaging setup, these specific features within the assemblies are barely distinguishable. Partially covering the tungsten sheet and overlapping one of the fuel pellet assemblies were two gas samples: krypton (2) and xenon ^24^(4). These gas samples were contained in 0.5-inch stainless steel tubes, pressurized to 90 and 95 psi, respectively. Swagelok SS316 fittings (5) secured the gas cells. Two scintillator samples, BaBrCl:Eu(0.1%), Au(0.1%)^33^(6), and CsPbBr$_3$:Eu(0.1%)^34^(7)-were each enclosed in separate 1-inch-diameter silica glass beakers. Lastly, a sodium chloride (NaCl) powder sample was stored in a 2-inch silica glass tube (8). To explore potential applications of this setup for future experiments, three small beads-borosilicate glass, zirconium, and tungsten carbide (9)-were embedded in the NaCl powder. These beads were tested to assess the feasibility of tracking their movement within molten salt. Although this setup is not designed for falling sphere measurements due to the significant density difference between tungsten carbide and NaCl, these initial tests provide valuable insights for future development.

For these large-FoV measurements, we employed the TRINIDI code, which requires defining two regions: an open-beam area and a uniformly dense region in the image. These regions were manually selected by defining ROIs around and within the samples in the image. Additionally, TRINIDI requires a list of materials that may be present in the image. The resulting reconstruction is depicted in Fig. 4, where each frame represents the density mapping of a specific material.

TRINIDI effectively identified most materials, generating visually consistent relative areal density distributions. Samples with significant resonances, such as tungsten, uranium, and the europium- and gold-containing scintillators, exhibited high signal-to-noise ratios with strong contrast. The pixel-by-pixel reconstruction enabled identification of textures and density variations within the samples, notably revealing the texture and separation of europium doping in solidified BaBrCl:EuAu, consistent with previous studies ^33^. Sodium chloride (NaCl) was identified through the broad resonance of Na-23 at 2.8 keV, which remained visible even for short path lengths due to its width. However, the high absorption cross-section of chlorine limited its distinguishability from other strong neutron-absorbing components. Additionally, the Swagelok SS316 fittings used for the gas tube caps were identified due to their composition, which includes 2% manganese and 2.5% molybdenum, both of which exhibit significant resonances in the measured energy range. TRINIDI exhibited remarkable sensitivity by clearly discerning a small tungsten carbide bead embedded within the salt column, despite its small size. Notably, no krypton (Kr) resonances were observed in the data. A follow-up investigation revealed that the gas pressure in the Kr tube was zero, likely due to unintentional evacuation during experiment preparation. As a result, Kr is not included in the results presented here.

Regarding isotopic quantification, spectral distortions caused by ZnS decay (as previously detailed) limited TRINIDI’s ability to yield meaningful isotopic density results within this NRI dataset. Nonetheless, this represents a significant achievement in near-fully automated reconstruction, requiring minimal user input or parameter tuning. Additionally, statistical limitations and high background levels may also impact the results. These results demonstrate the capability of this NRI setup-utilizing commonly available neutron scintillators such as $^{6}$LiF-ZnS:Ag-to differentiate and characterize various materials within a complex, multi-component sample, even in challenging scenarios where some elements exhibit overlapping or interfering signatures.

### Quantitative isotopic mapping

**Fig. 5:**
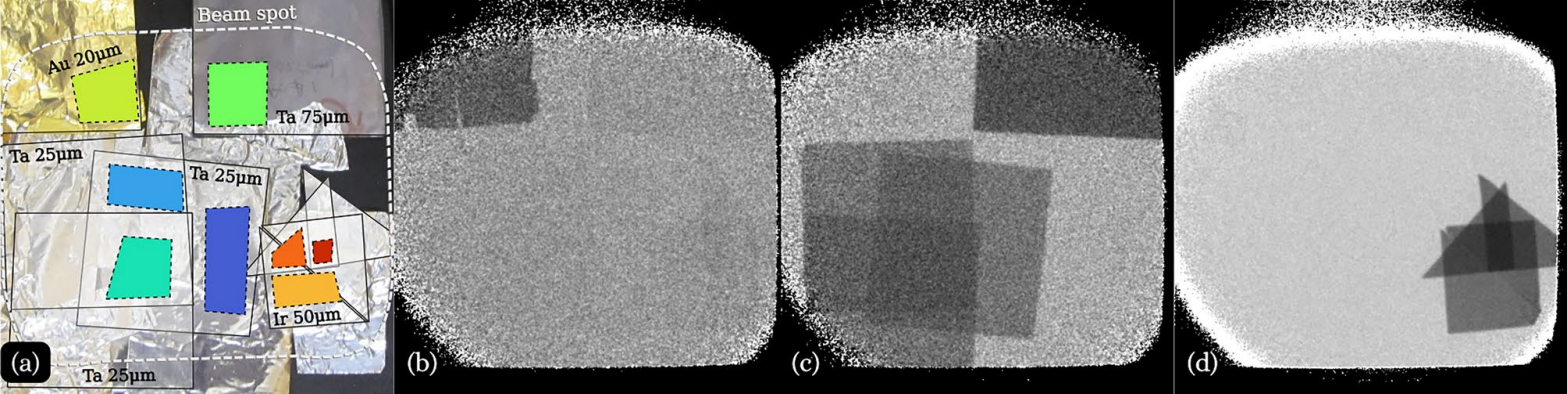
(**a**) Photograph of the sample alignment on the scintillation screen, with ROI for sample positions marked by dashed lines. Energy-window integrated transmission images captured around the neutron absorption resonance energies for (**b**) gold (Au-197) at 4.9 eV, (**c**) tantalum (Ta-181) at 4.28 eV, and (**d**) iridium (Ir-191) at 0.54 eV.

While remote qualitative material identification is a powerful and effective nondestructive testing and evaluation (NDTE) tool, quantifying materials with NRI has significant potential to advance material characterization techniques. To evaluate the effectiveness of this NRI setup in quantifying isotopic densities, we conducted measurements using thin foil samples of various materials, to assess the precision of sample thickness determination. The samples were simultaneously measured during a 7.5-hour run, while the open beam was measured for 10 hours. Transmission images were obtained by dividing the sample images by the corresponding open-beam images (see Section 2.5). Figure 5 illustrates the experimental setup and results of this neutron imaging study. Panel (a) shows the samples mounted on the aluminum backing of the scintillator with aluminum tape. Overlaid outlines indicate the positions of different foils within the FoV. The samples include iridium and tantalum foils with thicknesses of 50 and 25 µm, respectively, arranged in configurations of one to three layers, resulting in total thicknesses of up to 150 µm. Additionally, a 20 µm gold foil and a 75 µm tantalum foil are located at the top of the FoV. Panels (b)-(d) show three time-integrated transmission images corresponding to the principal resonances of gold, tantalum, and iridium. The pronounced resonance absorption in these images enables clear visual separation and identification of isotopic contributions. While the qualitative assessment of foil thickness variation through stacked layers is visually apparent, the critical question remains whether isotopic thickness measurements can be accurately derived from the distinct regions in these images.

**Fig. 6:**
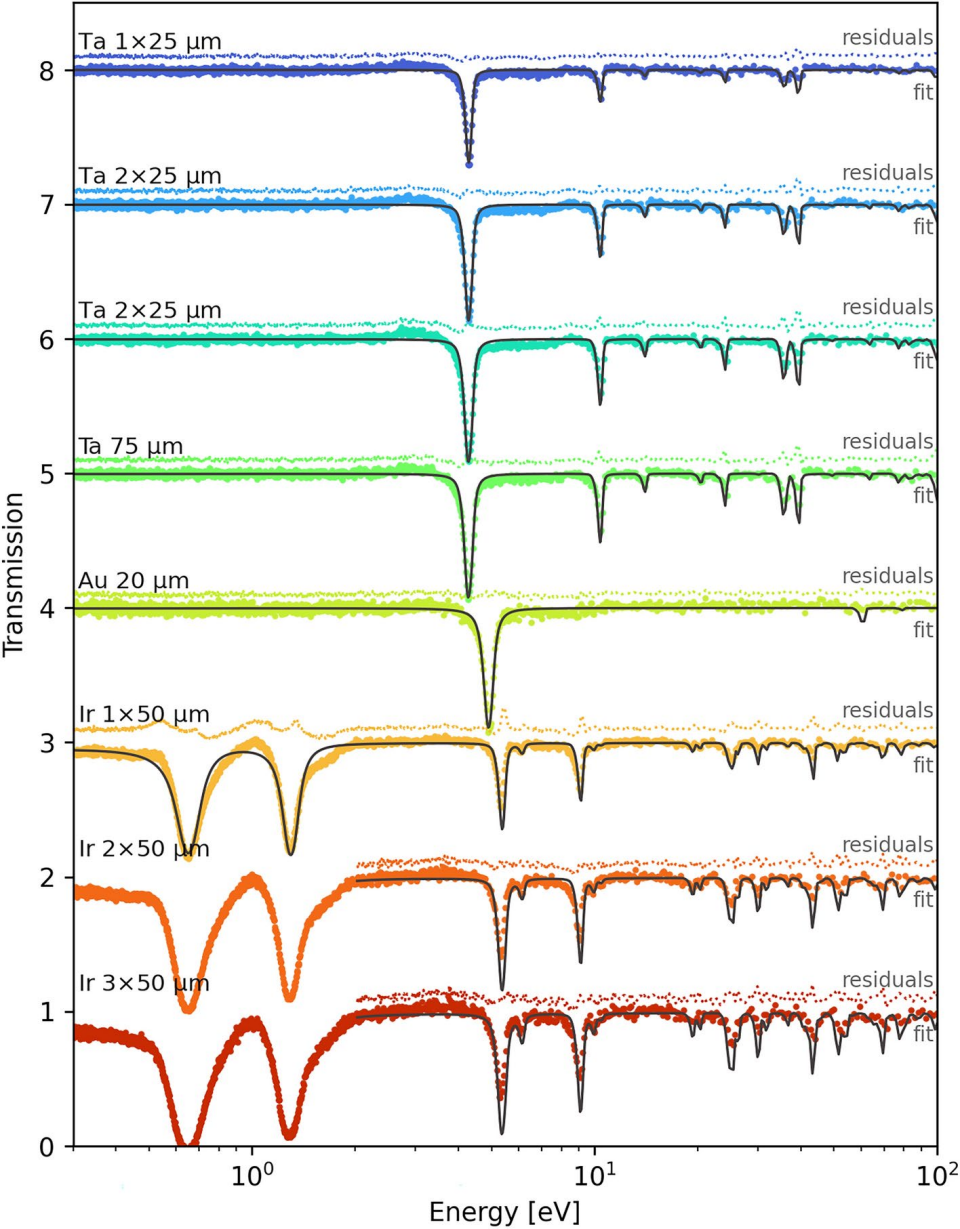
SAMMY fits of transmission data for stacked thin foil samples of various materials (Ta, Au, Ir) and thicknesses. Each fit is vertically offset by +1 from the previous one for clarity. The residuals (deviations from the fit) are shown as dotted lines above each respective fit. The color scheme corresponds to that used in Fig. 5, where different colors for the same material (e.g., Ir) represent different layer thicknesses. The fit parameters are listed in Table 2.

Due to the simplicity of fitting a limited of neutron transmission spectra, we used the PLEIADES and SAMMY codes to extract isotopic densities and determine sample thicknesses. We selected ROIs around the samples, each color-coded in panel (a) based on the fitting results presented later in Fig. 6. To achieve the required precision, we fitted each ROI using three free parameters: sample thickness, normalization, and a constant background. Sample thickness was determined by assuming the natural gravimetric densities of 19.3, 16.65, and 22.56 g/cm$^3$ for gold, tantalum, and iridium, respectively ^35^. Additionally, an empty ROI (containing no sample) was used to correct each TOF spectrum and account for temporal variations.

For the SAMMY fits, an energy range of 0.3 eV to 100 eV was used, except for the two thickest iridium foil samples, where a range of 2 eV to 100 eV was employed to exclude strongly saturated low-energy resonances that did not converge well. Figure 6 presents the SAMMY fit results, displaying transmission data for stacked thin foil samples of various materials (Ta, Au, Ir) and thicknesses. Each panel corresponds to a different foil sample and displays the fitted transmission curve (solid line) along with the corresponding residuals (deviations from the fit, plotted as dotted lines above each respective curve). For clarity, the panels are vertically offset by a constant value of +1 in the transmission scale.

While the theoretical curve aligns well with the experimental spectrum overall, notable distortions were observed, particularly at off-resonance energies. These distortions appear as undershoot or overshoot deviations from the expected resonance structure, most prominently at the base of several broad resonances. Notably, they are most evident at the first two resonances in the 50 µm-thick iridium layer sample. Furthermore, these discrepancies vanish when different scintillation materials, such as $^6$LiF-ZnO:Zn or $^6$Li-glass, are used. However, the origin of these distortions remain unclear and warrants further investigation.

The SAMMY fit results are presented in Table 2. Despite the spectral distortions, SAMMY successfully fit most of the data reasonably well and reconstructed the actual thickness for all eight samples. The predicted thickness is consistent with the measured foil thickness within uncertainties, with percentage differences below 5% in all cases. The background parameter, also listed in Table 2, remains consistent across all samples. These results demonstrate that $^6$LiF-ZnS:Ag can map isotopic areal densities with better than 5% accuracy, even without a precise prior background determination, despite its slower decay times compared to other scintillation screens.

**Table 2:** Comparison of actual and predicted thicknesses by SAMMY for different samples and number of layers.

Sample	Layers	Measured thickness [$\mu$m]	Predicted thickness [$\mu$m]	Thickness difference [%]	Predicted background [%]	Reduced $\chi ^2$
Ta	1	25	25.4±1.5	+1.5	24.7±2.4	1.6
	2	50	51.6±1.4	+3.2	24.6±1.6	1.6
	3	75	74.0±1.3	-1.3	24.3±1.0	3.6
	1	75	78.7±1.6	+4.9	24.4±1.1	2.0
Ir	1	50	50.3±0.5	+0.7	23.1±1.1	10.1
	2	100	101.8±4.7	+1.8	24.3±2.7	2.0
	3	150	153.4±5.8	+2.3	24.0±2.1	2.7
Au	1	20	18.9±0.8	-5.0	24.6±1.6	0.9

## Summary and outlook

This study demonstrates the first application of a large-FoV LumaCam setup, coupled with a $^{6}$LiF-ZnS:Ag scintillator, for epithermal neutron resonance imaging. We successfully imaged a diverse set of samples, including thick tungsten and tantalum foils for background estimation, thin-foil stacks for isotopic composition analysis, and a collection of irregularly shaped objects to demonstrate automatic reconstruction and isotope identification of complex scenes. The large-FoV setup enabled capturing of all samples in a single, streamlined acquisition, significantly reducing overall measurement time compared to previous lengthy, individual measurements.

The LumaCam system provides significant advantages due to its flexible and scalable design. Our current 120$\times$120 mm$^2$ setup maintains image quality across the demonstrated area, with infrastructure in place to support future expansion to 250$\times$250 mm$^2$ or larger. This scalability enables imaging of samples across various length scales and sizes. The large-FoV setup is particularly beneficial for heterogeneous samples with irregular shapes, allowing detailed, pixel-by-pixel analysis of specific regions within objects. The system’s versatility extends to its adaptability for various neutron applications (cold,thermal, epithermal, and fast) by changing the scintillator screen and adjusting optical magnification, eliminating the need for multiple dedicated systems.

The fully digital operation of the LumaCam enables post-acquisition optimization using various reconstruction algorithms, adapting to specific resolution or count-rate requirements. Event-by-event reconstruction facilitates neutron/gamma discrimination thus minimizing unwanted backgrounds in the final reconstructed image. A spatial resolution of approximately 340 $\mu$m was estimated for the large-FoV configuration. Although the Timepix3 sensor’s 256$\times$256 pixel array contributes to this resolution, the exact limiting factors for spatial resolution in this system require further investigation.

One of the primary challenges identified in this work relates to the use of $^6$LiF-ZnS:Ag as the scintillation screen. Its long decay components (5 and 80 µs) may introduce timing distortions in the measured neutron energy spectrum. Furthermore, the discrete and sparse sampling of photons during these extended decay curves affects time resolution in event reconstruction, potentially complicating resonance analysis. To address these limitations, we plan to explore alternative scintillators with faster decay times, such as $^6$Li-glass and $^6$LiF-ZnO:Zn, which are currently under investigation and have already demonstrated enhanced performance for epithermal neutron detection. Additional challenges include rate limitations and saturation effects. The Timepix3 sensor is limited to 80 MHit/s, which can pose challenges in high-gamma-background environments. Saturation effects were observed in the image intensifier at elevated count rates, especially following intense gamma bursts. To mitigate these issues, we implemented strategies such as placing lead bricks upstream and reducing optical apertures within the system.

Future improvements will focus on optimizing reconstruction algorithms and refining neutron event identification parameters. Additionally, future studies will explore the use of thicker scintillators for improved detection efficiency, which may require enhanced gamma suppression techniques. The next-generation TimePix4, featuring a larger 448$\times$512 stackable sensor, 0.2 nsec timestamp, and higher event rates, is expected to provide enhanced resolution across equivalent FoVs^36^.

In conclusion, the LumaCam system exhibits significant potential for high-precision, large-FoV epithermal NRI at spallation sources. While optimization opportunities remain, such as scintillator material selection, its versatility and cost-effectiveness make it particularly valuable for small- and medium-scale neutron facilities. The system’s adaptability,-enabled by interchangeable scintillator screens and flexible reconstruction algorithms-positions it as a multifunctional tool for diverse neutron measurement and imaging applications. Future enhancements will expand its capabilities, further advancing NRI across various applications.

## Data Availability

The datasets generated and/or analyzed during the current study are not publicly available due to their size, but are available from the corresponding author on reasonable request.
